# Dissociating the functions of superior and inferior parts of the left ventral occipito-temporal cortex during visual word and object processing

**DOI:** 10.1016/j.neuroimage.2019.06.003

**Published:** 2019-10-01

**Authors:** Philipp Ludersdorfer, Cathy J. Price, Keith J. Kawabata Duncan, Kristina DeDuck, Nicholas H. Neufeld, Mohamed L. Seghier

**Affiliations:** a Wellcome Centre for Human Neuroimaging, Institute of Neurology, University College London, London, UK; b Department of Cognitive Neuroscience, University of Tokyo, Tokyo, Japan; c Department of Neurology and Neurosurgery, McGill University, Montreal, Canada; d Department of Psychiatry, University of Toronto, Toronto, Canada; e Cognitive Neuroimaging Unit, Emirates College for Advanced Education (ECAE), Abu Dhabi, United Arab Emirates

**Keywords:** Connectivity, fMRI, Fusiform gyrus, Occipito-temporal sulcus, Reading and object recognition

## Abstract

During word and object recognition, extensive activation has consistently been observed in the left ventral occipito-temporal cortex (vOT), focused around the occipito-temporal sulcus (OTs). Previous studies have shown that there is a hierarchy of responses from posterior to anterior vOT regions (along the y-axis) that corresponds with increasing levels of recognition - from perceptual to semantic processing, respectively. In contrast, the functional differences between superior and inferior vOT responses (i.e. along the z-axis) have not yet been elucidated. To investigate, we conducted an extensive review of the literature and found that peak activation for reading varies by more than 1 cm in the z-axis. In addition, we investigated functional differences between superior and inferior parts of left vOT by analysing functional MRI data from 58 neurologically normal skilled readers performing 8 different visual processing tasks. We found that group activation in superior vOT was significantly more sensitive than inferior vOT to the type of task, with more superior vOT activation when participants were matching visual stimuli for their semantic or perceptual content than producing speech to the same stimuli. This functional difference along the z-axis was compared to existing boundaries between cytoarchitectonic areas around the OTs. In addition, using dynamic causal modelling, we show that connectivity from superior vOT to anterior vOT increased with semantic content during matching tasks but not during speaking tasks whereas connectivity from inferior vOT to anterior vOT was sensitive to semantic content for matching and speaking tasks. The finding of a functional dissociation between superior and inferior parts of vOT has implications for predicting deficits and response to rehabilitation for patients with partial damage to vOT following stroke or neurosurgery.

## 1 Introduction

Many functional imaging studies of reading have reported activation in the left ventral occipito-temporal (vOT) cortex, centred around the middle part of the left occipito-temporal sulcus. Because of its important role in reading, this region has often been referred to as the “visual word form area”. However, as the same region is activated during many tasks other than reading, it also appears to play a more general role in integrating visual inputs with the language system (see Price, 2012 for review). Given the size of the vOT region, and variability in where reading activation is reported, there are likely to be multiple subdivisions with dissociable functions. This has already been documented in the posterior-to-anterior direction (Y-axis) (Lerma-Usabiaga et al., 2018; Seghier and Price, 2011; Vinckier et al., 2007), and the lateral-medial direction (x-axis (Gao et al., 2017), but not in the inferior-superior direction. For example, posterior vOT is more sensitive than anterior vOT to stimulus-bound perceptual features while anterior vOT is more sensitive than posterior vOT to abstractions that support object recognition (Simons et al., 2003; Price and Mechelli, 2005; Levy et al., 2009; Seghier and Price, 2011). Although much less is known about functional divisions in the superior-to-inferior direction (Z-axis), high variability in the Z-axis has been seen in previous neuroimaging reviews (Jobard et al., 2003; Bolger et al., 2005; Taylor et al., 2013). The aim of current study was to investigate what drives variation in activation along the Z-axis (superior versus inferior).

In an extensive review of the literature (see Table 1), we found that reading activation localised to left vOT or the “visual word form area” varied 1.2 cm in the z-direction (from z = −8mm to z = −20mm). These differences have rarely been acknowledged or discussed (e.g. Nestor et al., 2012; Gauthier et al., 2000; James et al., 2005) but, in the bilingual literature, Bolger et al. (2005) reported activation peaks in inferior vOT for alphabetic writing and in superior vOT for logographic (Chinese and Japanese) writing. Given the contrasting nature of the visual word forms in alphabetic and logographic scripts, our first hypothesis is that superior and inferior vOT regions might be sensitive to different types of visual processing. Alternatively, our second hypothesis is that, because alphabetic scripts have closer links to phonological representations while logographic scripts have closer links to semantic representations, inferior vOT might have stronger connections to phonological regions and superior vOT might have stronger connections to semantic regions. A third hypothesis is that alphabetic and logographic scripts require different degrees of attention to detail and this might selectively influence activation in either the inferior or superior parts of vOT cortex.

**Table 1:** **Previous studies reporting left vOT activation during reading**: A MEDLINE search was conducted (from January 2000 to October 2018) using the keywords (i) ‘Reading’, (ii) ‘fMRI’ or ‘magnetic resonance imaging’ and (iii) ‘occipitotemporal’, ‘occipito-temporal’, or ‘visual word form area’ to identify papers that had reported activation during reading in left vOT. Relevant references within these articles also directed us to other papers that were considered in the literature review. Altogether, we identified 213 articles. We then excluded: (i) reviews and meta-analyses (i.e. those not reporting original-research), (ii) effects from subjects who were not neurologically or psychiatrically “normal” adults, or who had atypical learning, (iii) effects that were not related to visually presented words or pseudowords, (iv) effects not reported in standardized coordinates, (v) results of contrasts that compared visual stimuli to rest or fixation (because it was impossible to determine the level of cognitive processing that was driving activation), (vi) single case studies, (vii) co-ordinates related to laterality indices, (viii) effects in predefined regions of interest (region-based analyses), and (ix) studies published in non-English journals. Where appropriate, stereotactic Talairach coordinates were converted into Montreal Neurological Institute (MNI) space. For each study, we reported the location of the left vOT activation peak. The median of all vOT peaks is [x = −43 mm, y = −58 mm, z = −14.5 mm]. Activation contrasts were categorised as being related to: (1) changes in task demands where subjects performed different tasks with the same set of stimuli or (2) changes in stimulus demands where subjects performed the same task with different sets of stimuli. Task driven contrasts were further categorised into those primarily driven by visual (e.g. letter detection versus phoneme detection), semantic (e.g. semantic versus identity one-back matching), or general demands (e.g. one-back matching versus passive viewing). Stimulus driven contrasts were further categorised into those primarily driven by visual differences (e.g. written words versus pictures of objects), linguistic content (e.g. words versus false fonts), a combination of visual differences and linguistic content (e.g. words versus checkerboards), semantic content (e.g. high versus low imageable words), general demands (e.g. unfamiliar versus familiar words), or stimulus primes (i.e. less activation when stimuli were preceded by identical ones). In some papers, superior peaks at z  ≥  −12mm were labelled as inferior occipital gyrus instead of vOT.

Study	MNI coordinates			Factor driving activation
	x	y	z	
Cohen et al. (2002)	−39	−57	−9	Stimuli: visual/linguistic content
Binder et al. (2005a)	−42	−55	−10	Stimuli: general demands
Chee et al. (2000)*	−43	−56	−10	Task: semantic demands
Xu et al. (2015)	−40	−56	−10	Stimuli & task: visual/linguistic
Bruno et al. (2008)	−46	−56	−11	Stimuli: general demands
Xue et al. (2006)	−42	−53	−12	Task: general demands
Nosarti et al. (2010)	−44	−54	−12	Stimuli: general demands
Dehaene et al. (2010)	−45	−57	−12	Stimuli: primes
Stevens et al. (2017)	−52	−49	−13	Stimuli: visual content
Weiss and Booth (2017)	−36	−48	−14	Stimuli: general demands
Hayashi et al. (2014)	−48	−54	−14	Stimuli: visual/linguistic content
Purcell et al. (2011)	−40	−56	−14	Stimuli: visual/linguistic content

Quinn et al. (2017)	−42	−60	−8	Stimuli: general demands
Dehaene et al. (2004)	−44	−64	−8	Stimuli: primes
Peng et al. (2003)*	−43	−66	−9	Stimuli: linguistic content
Guo and Burgund (2010)*	−43	−70	−9	Task: visual demands
Schurz et al. (2010)	−44	−60	−10	Stimuli: general demands
Woollams et al. (2011)	−44	−62	−10	Stimuli: general demands
Weiss and Booth (2017)	−46	−62	−10	Stimuli: general demands
Cohen et al. (2008)	−42	−70	−10	Stimuli: linguistic content
Sussman et al. (2018)	−40	−62	−10	Stimuli: visual/linguistic content
Twomey et al. (2013)	−45	−58	−11	Stimuli: general demands
Kiehl et al. (1999)	−41	−60	−12	Stimuli & task: visual/linguistic
Carreiras et al. (2007)	−40	−66	−12	Stimuli: visual/linguistic content
Wimmer et al. (2016)	−48	−58	−14	Stimuli: general demands
Wright et al. (2008)	−48	−58	−14	Task: semantic demands

Yarkoni et al. (2008)*	−44	−52	−15	Stimuli: general demands
Mongelli et al. (2017)	−44	−55	−15	Stimuli: visual content
Cohen et al. (2002)	−42	−57	−15	Stimuli: visual/linguistic content
Sandak et al. (2004)	−45	−50	−16	Stimuli: semantic content
Richardson et al. (2011)	−40	−54	−16	Stimuli: linguistic content
Danelli et al. (2013)	−40	−56	−16	Stimuli: visual/linguistic content
Binder et al. (2005b)	−42	−52	−17	Stimuli: general demands
Kronbichler et al. (2004)	−42	−50	−18	Stimuli: general demands
Szwed et al. (2014)	−42	−54	−18	Stimuli: linguistic content
Schuster et al. (2015)	−39	−46	−20	Stimuli: visual/linguistic content
Dehaene et al. (2001)	−44	−52	−20	Stimuli: primes
Thesen et al. (2012)	−46	−52	−20	Stimuli: linguistic content

Chee et al. (2000)*	−43	−60	−15	Task: semantic demands
Kronbichler et al. (2009)	−48	−60	−15	Stimuli: general demands
Cohen et al. (2003)	−42	−63	−15	Stimuli: visual/linguistic content
Xue and Poldrack (2007)	−39	−66	−15	Stimuli: primes
Kao et al. (2010)	−41	−58	−16	Stimuli: linguistic content
Kherif et al. (2011)	−40	−58	−16	Stimuli: primes
Cohen et al. (2004)	−44	−64	−16	Task: visual demands
Mechelli et al. (2003)	−44	−64	−16	Stimuli: general demands
Wang et al. (2018)	−48	−64	−16	Stimuli: semantic content
Chee et al. (2003)*	−45	−58	−17	Stimuli: general demands
Booth et al. (2002)	−42	−60	−18	Stimuli: visual/linguistic content
Devlin et al. (2006)	−42	−60	−18	Stimuli: linguistic content
Kronbichler et al. (2007)	−48	−60	−18	Stimuli: general demands
Danelli et al. (2015)	−40	−64	−18	Stimuli: visual/linguistic content

**Fig. 1:**
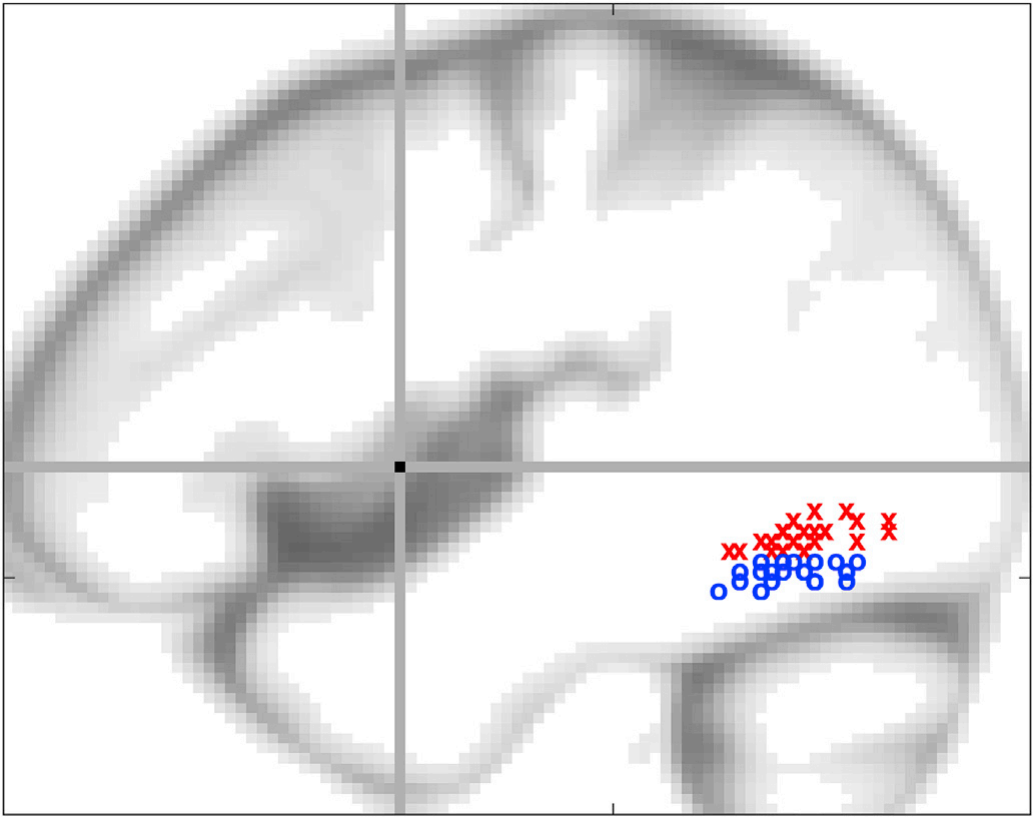
**Literature review.** This schematic figure illustrates the wide spatial variability of vOT localisation across functional imaging studies of reading. Each vOT peak represents the results of one of the selected 52 studies listed in Table 1: vOT coordinates above or below the median z = −15mm are shown in red (‘x’) or blue (‘o’) respectively. The y-axis (z = 0 mm) and the z-axis (y = 0 mm) are shown as grey lines, with their intersection at the AC point in black. The background of this figure is a sagittal view of the SPM's tissue probability map of CSF at x = −44mm.

Our literature review of vOT activation during reading studies did not identify clear evidence to support any of the above hypotheses. There were 52 studies that met our inclusion criteria and we split these studies according to whether they report (A) superior vOT activation that was less than 15 mm below the AC-PC line (z = 0 to −14) or (B) inferior vOT activation that was more than 14 mm below the AC-PC lines (z = −15 to z = −22). The functional border between superior and inferior vOT was set here to around −15mm (i.e. equivalent to the median of all reported z-coordinates across the selected 52 studies). This functional border ensured that half the foci (26/52) were located in superior vOT and the other half were located in inferior vOT, see Fig. 1. There was no corresponding division in the type of stimuli and tasks used by the study (see Table 1 for more details). We therefore investigated our own data to determine how superior and inferior vOT subregions respond when the demands on visual and semantic processing were varied by changing (i) the stimuli (pictures versus letters), (ii) their familiarity or semantic content (semantically meaningful or not) and (iii) the task (speaking versus matching). We also used dynamic causal modelling (DCM) to investigate condition-dependent connectivity to and from superior and inferior vOT subregions. Our rationale was motivated by previous studies that have shown convergence of different ventral and dorsal inputs to vOT (e.g. (Rauschecker et al., 2012; Rauschecker et al., 2011; Yeatman et al., 2012)).

## 2 Methods

This study was approved by the London Queen Square Research Ethics Committee. Full details of our experimental design and stimuli have been reported previously (Josse et al., 2008 (Seghier and Price, 2011); but the data have not previously been used to investigate visual and semantic effects in different parts of left vOT cortex.

### 2.1 Subjects

Fifty-eight right-handed healthy subjects participated in the functional MRI paradigm. All were native English speakers, had normal or corrected-to-normal vision, no history of neurological or psychiatric disorders and no awareness of developmental disorders (e.g. dyslexia), and gave written informed consent to participate in the study. Three different age groups were included: 20 teenagers aged 13–18 (11 female, 9 male), 24 young adults aged 19–33.7 years (13 female, 11 male) and 14 older adults aged 50–73.6 years (10 female, 4 male). Overall, there were 34 females and 24 males with a mean age of 30 years. All the teenagers had normal or above normal verbal and nonverbal IQ as measured using the Wechsler Intelligence Scale for Children (WISC-III). The effects of age and gender on behavioural and fMRI responses were investigated (see results) but did not explain our results. Three of the subjects were excluded from the connectivity analyses because they did not show activation in one of the regions of interest (*p* < 0.05 uncorrected in at least 5 voxels).

### 2.2 Experimental design

**Fig. 2:**
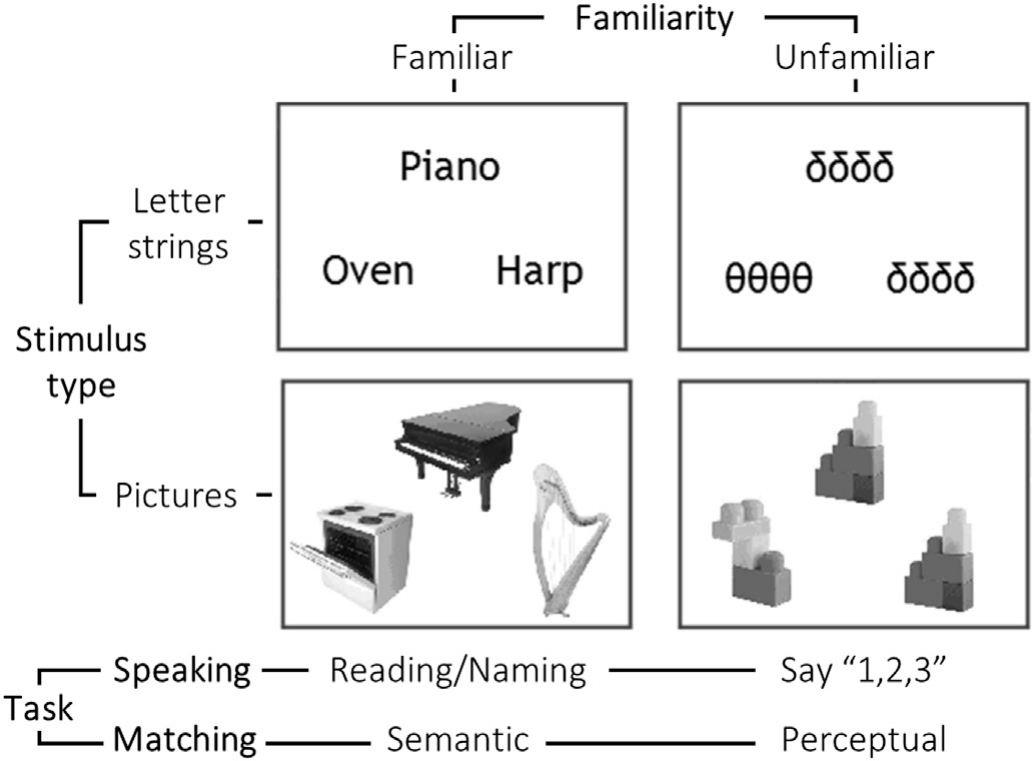
**Experimental design.** Our experimental paradigm manipulated the factors “stimulus type” (letter strings versus pictures), “familiarity” (familiar words and objects versus unfamiliar Greek letter strings and nonobjects) and “task” (matching versus speaking). In all trials, three stimuli were simultaneously presented as a “triad”, with one stimulus above and two stimuli below. In the matching tasks, participants made a finger press response to make: semantic matching decisions on words and objects (i.e. matching ‘Piano’ to ‘Harp’ rather than ‘Oven’) and perceptual matching decision on the unfamiliar stimuli (based on physical identity). In the speaking tasks, participants read/named aloud familiar words and objects and say “1,2,3” in response to seeing the unfamiliar stimuli.

There were four separate scanning sessions. In two sessions, subjects performed speaking tasks: reading aloud written words, naming aloud objects depicted in pictures, and saying “1,2,3” in response to seeing meaningless Greek letters and nonobjects. In the other two sessions (hereafter referred to as matching tasks), subjects made a finger press response to make semantic matching decisions on written words and pictures of objects as well as perceptual matching decisions on Greek letters and nonobjects. None of our subjects spoke Greek and our tasks discouraged subjects from associating Greek letters and specific (i.e. mathematical) concepts. The same stimuli were used across different sessions. Within each session, there were (i) 4 blocks of written words, (ii) 4 blocks of pictures of objects, (iii) 2 blocks of meaningless Greek letter strings, (iv) 2 blocks of meaningless pictures of nonobjects and (iv) 6 blocks of fixation. The order of conditions was counter-balanced within and across sessions. The experimental design is summarised in Fig. 2.

### 2.3 Stimuli

Stimuli were presented in the scanner by a video projector, a front-projection screen and a system of mirrors fastened to the MRI head coil. All stimuli were presented in same-format triads with one item above two other items (see Fig. 2). In the semantic and perceptual matching tasks, the item above was the target and the two items below provided the matching and non-matching choices that the subject was required to select from. In the speaking tasks, subjects were required to first attend (i.e. read, name, or say “123”) to the item above followed by attending to the lower left and then lower right items. Each triad remained on the screen for 4.32 s, followed by 180 ms of fixation. There were four stimulus triads of the same type per block (18 s per block) and each block was preceded by 3.6 s of instructions to indicate the type of response required. Fixation periods of 14.4 s length were interleaved after every two stimulus blocks. Across the experiment, subjects were presented with a total of 192 pictures of objects and the 192 corresponding 3–6 letter written names of objects (i.e. the semantic content and names were matched in the picture and word conditions). Words that were read were presented as pictures in the semantic matching condition and pictures that were named were presented as words in the semantic matching condition.

### 2.4 MRI acquisition

The experiment was performed on a 1.5-T Siemens system (Siemens Medical Systems, Erlangen, Germany). In the functional MRI (fMRI) sessions, imaging consisted of a single shot gradient Echo Planar Imaging (EPI) sequence (repetition time/echo time/flip angle = 3600 ms/50 ms/90°, field of view = 192 mm, matrix = 64 × 64, 40 axial slices, 2 mm thick with a 1-mm gap). Functional scanning was always preceded by 14.4 s of dummy scans to ensure tissue steady-state magnetization. A generalised reconstruction algorithm was used for data pre-processing to avoid ghost-EPI artefacts. An anatomical scan was also acquired for each subject and used for spatial normalisation. This was a 3D T1-weighted, modified equilibrium Fourier transform sequence with the following parameters: TR = 12.24 ms, TE = 3.56 ms, TI = 530 ms, FOV = 256 mm × 224 mm, acquisition matrix = 256 × 224, 1 mm slice thickness for 1 mm isotropic voxels.

### 2.5 Functional MRI data processing and analysis

Image processing and statistical analyses were performed using standard procedures in SPM (Wellcome Trust Centre for Neuroimaging, London, United Kingdom, http://www.fil.ion.ucl.ac.uk/spm/). All functional volumes were spatially realigned, un-warped, normalised to MNI space using the unified normalisation-segmentation procedure. The normalised volumes were written out at voxels size of 2 × 2 × 2 mm. These volumes were smoothed with an isotropic 6 mm full width at half maximum (FWHM) Gaussian kernel to compensate for residual anatomical variability and to permit application of Gaussian random-field theory for statistical inference (Friston et al., 1994).

Statistical analyses of the functional data were performed within a mixed effect model framework. In the subject-specific first-level models, the pre-processed functional volumes were submitted to a fixed-effects analysis using the general linear model at each voxel. Each stimulus onset was modelled as an event in condition-specific “stick-functions” lasting 4.32 s per trial and having a stimulus onset interval of 4.5 s. The resulting stimulus functions were convolved with a canonical hemodynamic response function that provided regressors for the general linear model. For each task condition we included 3 regressors: instruction, correct trials, and incorrect trials. Time-series from each voxel were high-pass filtered (1/128-Hz cut-off) to remove low-frequency noise and signal drift. From each subject's first level analysis, we extracted eight contrast images (one for each of the eight conditions' correct trials relative to fixation). These subject-specific images were then used for the second-level random-effects group analysis. This was comprised of a repeated measures ANOVA with eight conditions corresponding to the eight first level contrast images (see below).

### 2.6 Activation effects of interest

This paper is only concerned with effects located along the left occipito-temporal sulcus (OTs) that is known to be an important reading area (and often referred to as the visual word form area), see Fig. 1. The OTs is present in 100% of adults (Malikovic et al., 2012). In the y axis, OTs extends anteriorly to y = −45 mm and posteriorly to y = −75 mm, an extent that covered the range of potential vOT localisation in healthy adults (Cachia et al., 2018). The mid-point is therefore y = −60mm, extending 15 mm anterior and 15 mm posteriorly. In the z direction, the mid-point was located at z = −15mm, because this point corresponds to: (i) the z-coordinate at which the probabilities of being in cytoarchitectonic regions FG2 or FG4 are equal at y = −60mm (Lorenz et al., 2017; Weiner et al., 2017), according to Eickhoff et al. (2005), (ii) the median of the z-coordinate in our extensive literature review (Table 1), and (iii) the midpoint between the most superior (z = −10) and most inferior (z = −20) parts of the occipito-temporal sulcus at y = −60 mm. In the x direction, our ROI is located between x = −38 and x = −46 mm. Considering x, y and z co-ordingates together, the mid-point of our vOT ROI was set at [-42 -60, −15].

Within this region, we report main effects of task (i.e. speaking versus matching tasks), familiarity (i.e. familiar words and objects versus unfamiliar nonobjects and letter strings), and stimulus type (i.e. pictures versus letter strings). The comparison between familiar/meaningful stimuli and unfamiliar/meaningless stimuli (i.e. the familiarity effect) also reflects the semantic content of our stimuli. In addition, we report interactions between these variables when significant and the main effect of all conditions > fixation where there was also significant activation for each of the 8 conditions compared to rest at a statistical threshold of p < 0.001 uncorrected (this was achieved using the inclusive masking option in SPM). Correction for multiple comparisons was based on a sphere of 15 mm radius centred on this mid-point. In the x and z dimensions, this sphere conservatively included areas beyond the occipito-temporal sulcus, to ensure that we did not impose harsh or false boundaries on any apparent function subdivisions. We report the spatial location of different effects along the y-axis and the z-axis with reference to the current probabilistic definitions of cytoarchitectonic areas of the Anatomy toolbox in SPM (Eickhoff et al., 2005).

The anatomical definition used here was in standard MNI co-ordinates, after spatial normalisation, because we wanted to establish findings that generalised over groups of subjects, as in most group level functional neuroimaging studies. Alternatively, this might not be possible given potential variability from subject to subject in the size, shape, location and depth of the occipito-temporal sulcus (Kim et al., 2008; Ma et al., 2011; Ono et al., 1990) Malikovic et al., 2012), as well as in the number of its segments, side branches and cytoarchitectonic zones/areas (Caspers et al., 2013). For instance, Destrieux et al. (2010) have shown that the (lateral) occipito-temporal sulcus is discontinuous and difficult to differentiate, and it can connect to surrounding sulci including the anterior collateral transverse sulcus, the anterior occipital sulcus, or the inferior temporal sulcus (Destrieux et al., 2010).

### 2.7 Dynamic causal modelling

**Fig. 3:**
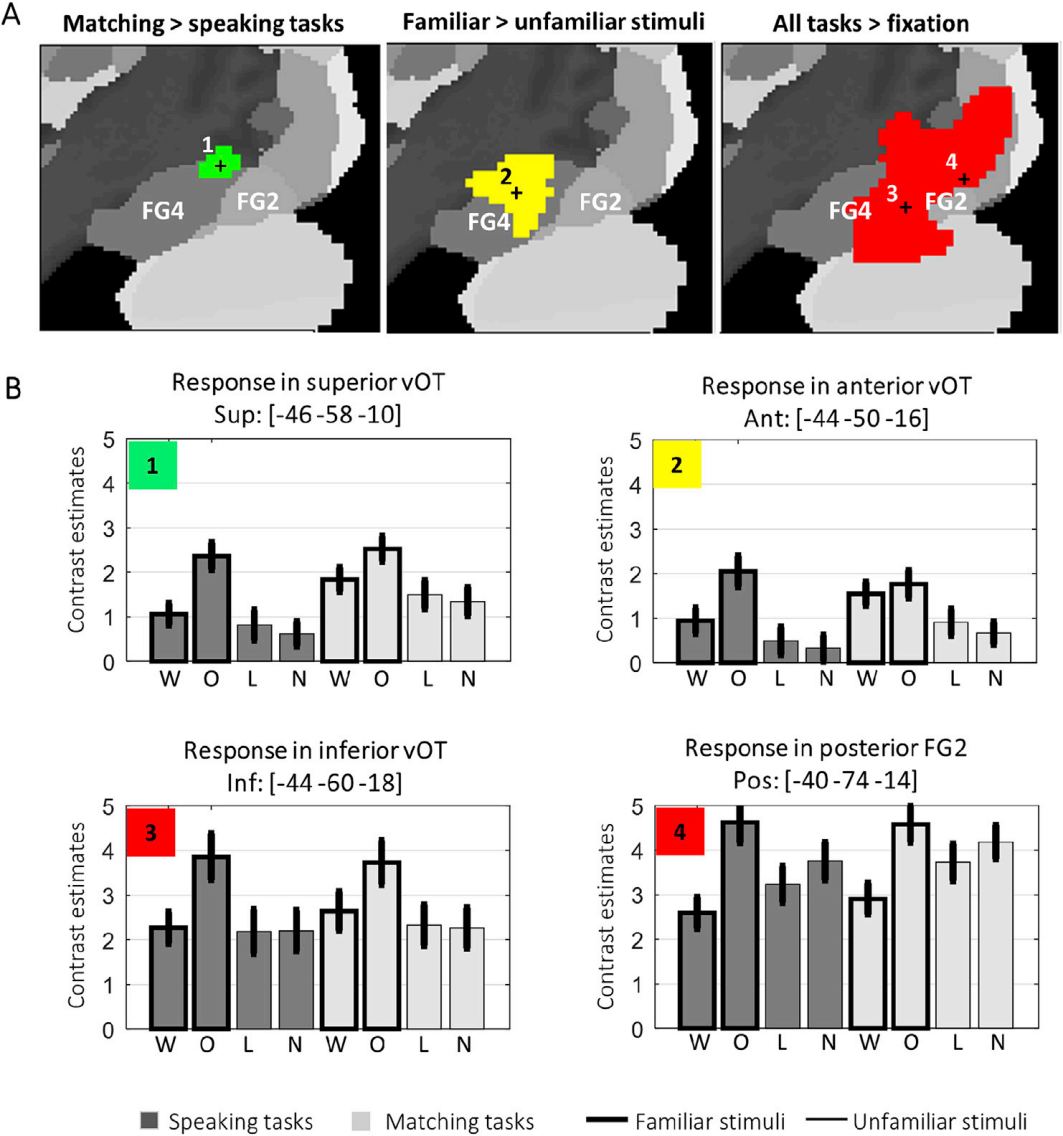
**Activation findings.** (A) Activation for matching versus speaking tasks (green), familiar versus unfamiliar stimuli (yellow), and all tasks versus fixation baseline (red), projected on a sagittal view (at x ​= ​−44mm) of the Anatomy Toolbox's cytoarchitectonic maps in MNI space. (B) Brain activation (effect size) for all eight task conditions at the peak voxels for: the task effect in superior vOT [-46, −58, −10]; the familiarity effect in anterior vOT [-44, −50,-16], and all conditions compared to fixation in inferior vOT [-44,-60,-18] and posterior fusiform [-40, −74, −14]. The bars report average activation estimates with error bars indicating 90% confidence intervals. Abbreviations: W ​= ​words, O ​= ​objects, L = (Greek) letters, N ​= ​nonobjects, FG2 and FG4 ​= ​cytoarchitectonic areas of the Anatomy Toolbox, ‘+’ ​= ​location of the peaks of interest used in the plots below.

We used dynamic causal modelling (DCM; Friston et al., 2003) to investigate neuronal interactions (i.e. effective connectivity) among four different regions in the occipito-temporal sulcus that were each observed to be activated in our voxel-based analyses (see Fig. 3 and Section 3.3). DCM analyses were carried out using DCM12 as implemented in SPM12. In brief, DCM estimates three sets of parameters: 1) input/extrinsic parameters that quantify how brain regions respond to external stimuli, 2) endogenous parameters reflecting the latent connectivity that characterizes the context-independent coupling between regions, and 3) modulatory parameters that measure changes in effective connectivity induced by experimental conditions.

In our case the input was modelled as a regressor representing all task conditions of the session. Therefore, the endogenous parameters reflect the average connectivity during the matching or speaking tasks. We were also interested in the modulatory effects of familiar words and objects. These parameters reflect connectivity changes over and above that for the unfamiliar items (i.e. Greek letter strings and pictures of nonobjects). Finally, we also tested for differences between words and pictures of objects.

All connectivity parameters are expressed in units of Hertz within the DCM framework. Positive values indicate that, as activity increases in the originating region, the rate of change in activity in the target region also increases. Negative values indicate the reverse (i.e. as activity increases in the originating region, a decrease in the rate of change in activity occurs in the target region). All parameters (endogenous and modulatory) of the DCM model and their posterior probabilities were then assessed with Bayesian inversion by means of an expectation-maximization algorithm (Friston et al., 2003). More details about DCM can be found elsewhere (Friston et al., 2003; Seghier et al., 2010; Stephan et al., 2010).

#### 2.7.1 Data extraction

To conduct the DCM analyses, we extracted the summarised time-series (i.e. principal eigenvariates) for each of the four regions of interest (ROI) for each separate experimental session from each first level analysis. The extracted ROI time-series were adjusted based on a subject-specific F-contrast that retained the experimental effects of interest (i.e. correct trials of all 8 conditions) and regressed out variance caused by factors of no interest (i.e. incorrect trials, instructions). The time-series were then separately concatenated for (a) the two speaking task sessions and (b) the two matching task sessions and entered into the DCM analyses.

#### 2.7.2 Parameter estimation

The exact mechanisms behind the differential activation responses that we observed were unknown. It was therefore important to specify a range of alternative models and search for useful models in the model space (Richardson et al., 2011; Seghier and Price, 2011). In the current study we did not have strong prior expectations about how our regions would interact. Since there are thousands of possible models that can be constructed with four ROIs, estimating the parameters of all models would be computationally inefficient. We therefore used Bayesian Model Reduction (BMR) which involves only estimating a full or parent model, containing all the parameters of interest and then use the posterior estimates of this full model to derive the posterior estimates (and model evidence) of reduced models, in which one or more parameters are systematically removed. BMR gives similar results as the standard approach but is more computationally efficient (Rosa et al., 2012). In our case the full model included input to the most posterior region and full connections (forward and backward) between all regions with the exception of any direct connections between the input region and the most anterior vOT region (i.e. visual information was assumed to initially flow forward from posterior vOT regions). Using the BMR approach, the connectivity parameters are estimated by taking a weighted average of the parameters under each model (Rosa et al., 2012). This approach, called Bayesian Model Averaging (BMA), accommodates uncertainty about the underlying model structure on the parameter estimates.

#### 2.7.3 Connectivity effects of interest

At the group level, we used one-sample t-tests to evaluate whether a parameter (i.e. an endogenous or modulatory connection) is non-zero. We were also interested in modulatory connections (i.e. connectivity changes for familiar words and objects over and above that for the unfamiliar items). This allowed us to assess condition-dependent connectivity to and from superior and inferior vOT subregions.

## 3 Results

### 3.1 In-scanner behaviour

Mean accuracy for all 8 tasks was above 90% and lowest accuracy was above 75% for all 8 tasks (see Table 2). fMRI activation (below) is reported for correct trials only. Matching response times were significantly slower (p < 0.05, 2 tailed *t*-test) for the 14 older participants (50–74) than the 44 younger participants (13–34) but there was no significant difference in response times for the teenagers (13–18) and young adults (19–34).

**Table 2:** **In-scanner accuracy and response times** Mean accuracy (and standard deviation) are reported for all 8 tasks. Response times were only available for the matching tasks (post-decision finger press speed) but not for the speaking tasks due to difficulties extracting voice onset from the noise of the scanner.

Condition		Accuracy (%)				Response times in seconds			
*Task*	*Stimuli*	*Mean*	*Lowest*	*Highest*	*SD*	*Mean*	*Lowest*	*Highest*	*SD*
Matching	Objects	93.5	75	100	4.5	1.7	1.3	2.6	0.3
	Words	90.3	81	100	5.4	1.8	1.2	2.3	0.3
	Non-objects	98	81	100	3.4	1.1	0.7	1.8	0.2
	Greek letters	99.1	88	100	3.0	1.1	0.7	1.7	0.2
Speaking	Objects	99.8	83	100	0.9	not available			
	Words	96.2	94	100	3.8				
Say “1,2,3”	Nonobjects	100	100	100	0				
	Greek letters	100	100	100	0				

### 3.2 Activation results

#### 3.2.1 The main effect of task: matching vs. speaking

The voxel-wise analysis identified a main effect of task with higher activation for matching than speaking tasks at MNI coordinates [-46 -58 -10] with a Z score of 4.2 (p = 0.004 corrected for multiple comparisons within a region of 15 mm radius centred on [-42 -60 -14] the mid-point of the occipito-temporal sulcus (see Section 2.6). This effect was located at the most superior edge of the middle part of the occipito-temporal sulcus, with a high probability of being within area FG4, according to (Lorenz et al., 2017; Weiner et al., 2017), see Fig. 3A. Activation in this superior vOT region was higher for matching than speaking tasks when the stimuli were words (Z = 4.6) and unfamiliar stimuli (Nonobjects: Z = 3.4; Greek letters: Z = 3.2) but not objects (Z < 1) that strongly activated superior vOT irrespective of task (Fig. 3B). This resulted in a weak task by semantics by stimulus type interaction (Z scores = 2.45, p < 0.01).

Greater activation for matching than speaking in this superior vOT region [-46 -58 -10] was observed for each of the three age-groups tested (p < 0.05 for 20 teenagers, 24 young adults and 14 older adults) and for both males and females (p < 0.05 for both) There was no significant difference between the size of the effect for any pair of participant group (p > 0.30, two tailed t-tests).

There was no significant effect (p > 0.05 uncorrected) in the opposite direction (i.e. speaking more than matching tasks) anywhere in our ROI along the occipito-temporal sulcus (from y = −75 to y = −45).

#### 3.2.2 The main effect of stimulus familiarity: familiar > unfamiliar stimuli

The voxel-wise analysis showed a main effect of stimulus familiarity with higher activation for familiar (words and objects) than unfamiliar stimuli (Greek letters and non-objects) at the anterior end of OTs (anterior vOT). Peak activation was observed at [-44 -50 -16] (Z > 8), and extended superiorly (Z score >8.0 at [-44 -50 -8]) and inferiorly (Z score = 7.8 at [-36 -42 -24]) even when the statistical threshold (p < 0.05) was corrected for multiple comparisons across whole brain, and limited to voxels where the effect of semantics was significant for both letter strings (Z = 6.0) and pictures (Z > 8). Both inferior and superior regions fell within the cytoarchitectonic area FG4, see Fig. 3A. Therefore, there was no evidence for a distinction between superior and inferior parts of anterior vOT in either our data or cytoarchitecture.

#### 3.2.3 The effect of stimulus type (letter strings versus picture)

There were no parts of our ROI that were more activated for pictures of nonobjects compared to Greek letters (or vice versa), even when the threshold was reduced to p < 0.05 uncorrected. However, stimulus type interacted with semantic content in all regions of interest (p < 0.001) because activation was highest for objects (pictures with semantic content) than all other conditions (see Fig. 3B).

#### 3.2.4 The effect of all stimuli more than rest

All conditions (relative to fixation) activated extensive regions of the occipital cortex (see Fig. 3) that included activation at both inferior and superior ends of OTs. Peak activation (across conditions) was identified in the posterior part of cytoarchitectonic area FG2, at the tail end of OTs at [-40, −74, −14] with a Z score of 9.5.

#### 3.2.5 Differences in the response properties of superior, inferior and anterior vOT regions

To compare the response properties of the superior, inferior and anterior vOT regions, we extracted subject specific responses for each condition in each of these regions and tested for region by condition interactions using IBM SPSS Statistics for Windows, Version 22.0 (Armonk, NY: IBM Corp). The effect of interest was Region, and how this interacted with the effect of task, familiarity and stimulus type in a 2x2x2x2 repeated measures ANOVA). This was repeated when the regions were superior and inferior vOT and when the regions were superior and anterior vOT.

Superior vOT responses were extracted at the peak co-ordinates for matching more than speaking [-46 -58 -10]. Inferior vOT was positioned at [-44 -60 -18] after searching for the voxel with the highest activation for all conditions relative to fixation, within 4 mm of [x = −46, y = −58, z = −20] – the most inferior part of the occipito-temporal sulcus directly beneath the superior vOT region. Anterior vOT responses were extracted at the peak co-ordinates for familiar more than unfamiliar stimuli [-44 -50 -16].

When the superior and inferior vOT responses were compared, there was (i) a main effect of region (p < 0.0001) because responses were higher in inferior than superior vOT; and (ii) an interaction between region and task (p = 0.009) because the effect of task was significantly greater in superior than inferior vOT. Region did not interact with familiarity or stimulus type and there were no three way or four way interactions (p > 0.05).

When the superior and anterior vOT responses were compared, there was (i) a main effect of region (p = 0.002) because responses were higher in superior than anterior vOT; (ii) an interaction between region and task (p = 0.01) because the effect of task was higher in superior than anterior vOT, and (iii) an interaction between region, familiarity and stimulus type (p = 0.046) because the effect of semantics on words was greater in anterior than superior vOT. There were no other significant interactions with region (p > 0.05).

### 3.3 Connectivity results

Having identified different functional vOT subdivisions in our voxel based analysis, we tested whether functional differences would also be observed in how superior and inferior parts of vOT interacted with posterior and anterior areas. Specifically we compared effective connectivity in two different pathways: a superior vOT pathway and an inferior vOT pathway. For both pathways, the input area was the posterior FG2 area [-40, −74, −14] where peak activation was observed for all stimuli compared to fixation (see Section 3.2.4 above) and the end point of the pathway was the anterior vOT area (i.e. FG4) [-44, −50, −16] where peak activation was observed for familiar compared to unfamiliar stimuli. Connections between these two regions were routed via either (i) the superior vOT area [-46, −58, −10] (i.e. posterior-superior FG4) identified for the main effect of matching more than speaking, or (ii) the inferior vOT area [-44,-60, −18] (i.e. anterior-inferior FG2) identified in Section 3.2.5.

A full report of the DCM findings can be found in Table 3. Here we highlight the results that distinguish the functions of the superior and inferior pathways.

**Table 3:** **Connection strengths (in Hz):** Strength of endogenous and modulatory connections during matching (A) and speaking (B) tasks. Abbreviations: Pos = input region in posterior FG2, Sup = superior (middle) vOT (posterior-superior FG4), Inf = inferior (middle) vOT (anterior-inferior FG2), Ant = anterior vOT (FG4).

A. Matching tasks													
Connection		Endogenous				Semantic > perceptual matching				Objects > Words			
From	To	M	SD	*t* (53)	*p*	M	SD	*t* (53)	*p*	M	SD	*t* (53)	*p*
Pos	Sup	0.21	0.02	12.83	<.001*	0.27	0.03	8.45	<.001*	0.03	0.03	0.86	>.2
	Inf	0.27	0.02	12.56	<.001*	0.34	0.04	8.43	<.001*	0.06	0.03	1.96	= .056
Sup	Pos	0.02	0.04	0.30	>.2	−0.08	0.04	−2.04	= ​.047*	0.04	0.04	0.75	>.2
	Inf	−0.13	0.02	−5.83	<.001*	−0.10	0.04	−2.65	= ​.011*	−0.12	0.04	−3.47	= ​.001*
	Ant	0.04	0.02	1.31	= .197	**0.12**	**0.02**	**4.96**	< .001*	−0.02	0.02	−1.01	>.2
Inf	Pos	0.09	0.04	2.41	= ​.020*	−0.13	0.02	−5.46	<.001*	0.09	0.03	2.68	= ​.010*
	Sup	−0.18	0.01	−13.95	<.001*	−0.06	0.04	−1.81	= .076	−0.05	0.03	−1.66	= .104
	Ant	0.09	0.02	4.93	<.001*	**0.07**	**0.02**	**3.22**	= .002*	0.01	0.02	0.32	>.2
Ant	Sup	0.09	0.02	5.31	<.001*	−0.21	0.03	−6.96	<.001*	−0.10	0.03	−3.09	= ​.003*
	Inf	0.00	0.04	−0.15	>.2	−0.27	0.04	−7.11	<.001*	−0.13	0.02	−7.09	<.001*

B. Speaking tasks																
Connection			Endogenous			Reading/Naming > saying “1,2,3″						Objects > Words				
From	To	M	SD	*t* (53)	*p*	M	SD		*t* (53)	*p*		M		SD	*t* (53)	*p*
Pos	Sup	0.17	0.01	21.74	<.001*	0.17		0.03	6.14	<.001*			0.13	0.02	5.16	<.001*
	Inf	0.27	0.02	14.51	<.001*	0.31		0.02	12.79	<.001*			0.15	0.03	5.56	<.001*
Sup	Pos	0.25	0.08	3.09	= ​.003*	−0.07		0.04	−1.88	= .066			0.02	0.04	0.36	>.2
	Inf	−0.02	0.04	−0.66	>.2	−0.14		0.04	−4.00	<.001*			−0.08	0.03	−2.62	= ​.012*
	Ant	0.22	0.03	6.85	<.001*	**0.03**		**0.02**	**1.01**	> **.2**			0.02	0.03	0.57	>.2
Inf	Pos	−0.07	0.04	−1.96	= .056	−0.08		0.03	−2.57	= ​.013*			0.16	0.02	7.09	<.001*
	Sup	−0.18	0.01	−16.75	<.001*	−0.01		0.03	−0.59	>.2			−0.10	0.03	−3.80	<.001*
	Ant	−0.01	0.01	−1.92	= .060	**0.09**		**0.02**	**5.21**	< .001*			0.01	0.01	−0.11	>.2
Ant	Sup	0.10	0.02	5.21	<.001*	−0.21		0.03	−6.86	<.001*			−0.07	0.02	−3.22	= ​.002*
	Inf	0.00	0.02	−0.37	>.2	−0.21		0.02	−12.13		<.001*		−0.29	0.03	−9.49	<.001*

#### 3.3.1 Average connectivity

Over all stimuli, the strength of connectivity was (i) significantly stronger in the superior pathway than the inferior pathway for speaking (t = 5.53, p < 0.001), with (ii) a trend for the opposite direction (more for the inferior pathway than the superior pathway) for matching (t = 1.83, p = 0.07). This resulted in a significant pathway (superior versus inferior vOT) by task (matching versus speaking) interaction (F (54) = 21.86, p < 0.001).

#### 3.3.2 Semantic versus unfamiliar stimuli

**Fig. 4:**
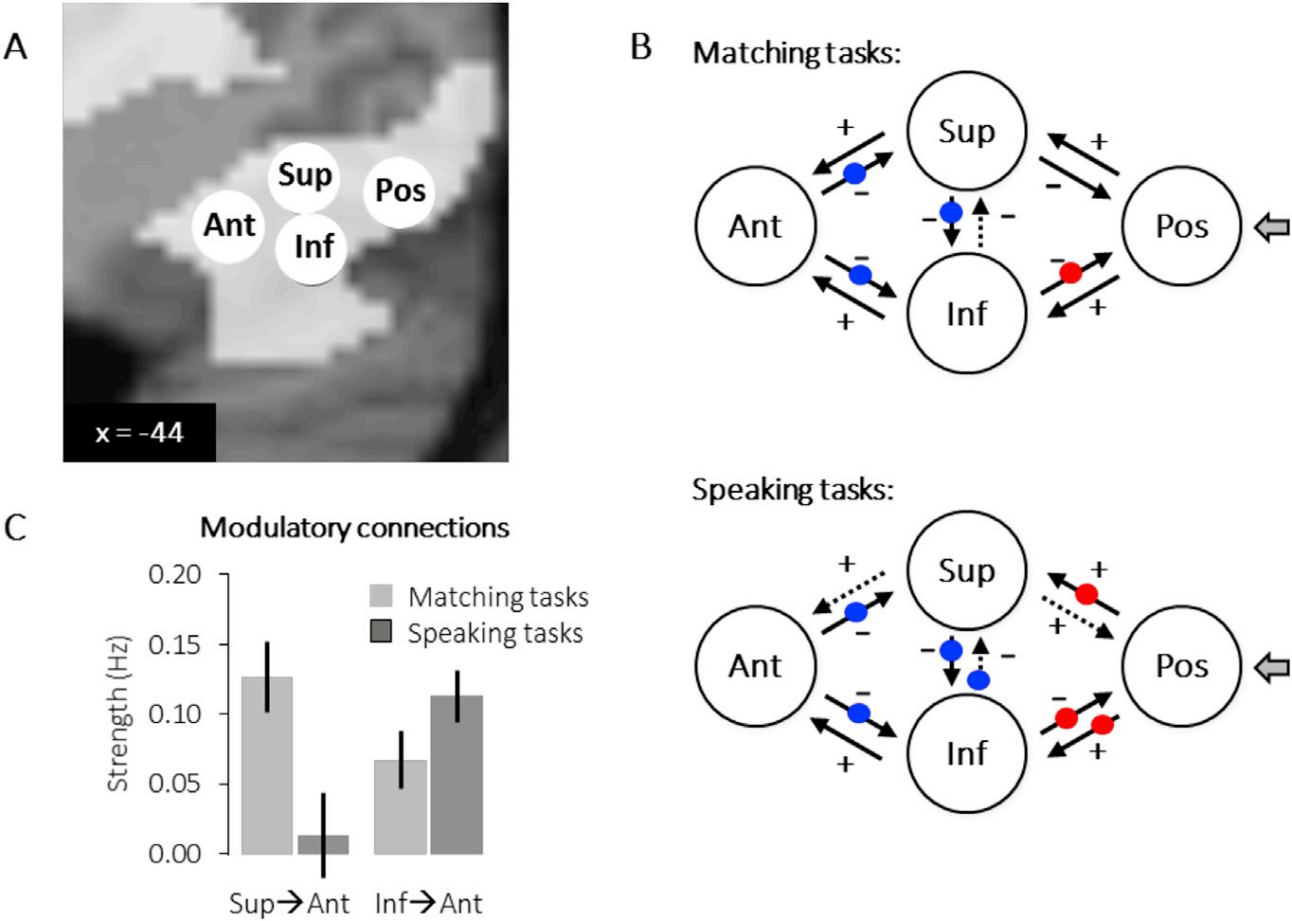
**Connectivity findings.** (A) Localisation of regions of interest projected onto a sagittal view of a canonical structural brain image. Additionally, activation for reading versus fixation baseline is shown in white. Abbreviations: Ant ​= ​anterior vOT, Sup ​= ​superior posterior vOT, Inf ​= ​inferior posterior vOT, Pos ​= ​posterior input region. (B) Modulatory (words and objects ​> ​unfamiliar stimuli) connections between the four regions of interest included in the dynamic causal modelling (DCM) analysis. Solid lines: significant modulations (p ​< ​0.05), dashed lines: no significant modulations; plus ‘+’ sign: positive modulations; minus ‘-’ sign: negative modulations; blue dots: stronger modulations for word than picture stimuli; red dots: stronger modulations for picture than word stimuli (see Table 3 for a list of all effects). (C) Task by connection interaction. Bars represent average modulatory connection strengths (in Hz) from superior to anterior vOT and from inferior to anterior vOT during the matching versus the speaking tasks. Error bars represent ± 1 standard error of the mean.

Connectivity in the inferior pathway increased with semantic content for both the matching and the speaking tasks (Fig. 4). In the superior pathway, connectivity increased for semantic content during matching tasks but not during speaking tasks (Table 3). Consequently, during the speaking tasks, the effect of semantic content was less in the superior pathway than the inferior pathway (t = 2.3, p < 0.05). This resulted in a significant pathway (superior versus inferior) by task (matching versus speaking) interaction for the modulatory effects (F (54) = 6.55, p = 0.014).

## 4 Discussion

This study investigated whether activation and connectivity differed, according to experimental task or stimuli, in superior and inferior parts of ventral occipito-temporal (vOT) cortex. An extensive review of the literature did not generate any clear hypotheses for a functional dissociation in these regions although it was clear that the co-ordinates of peak vOT activation vary considerably in the Z-axis (see Table 1). Our aim was to identify which task and stimulus variables influenced activation in the z-axis while excluding explanations of this variability in terms of random inter-subject variability in functional anatomy or insufficient spatial resolution in fMRI data.

To search for functional differences in the response of superior and inferior vOT subregions, we tested for effects of task (matching versus speech production), familiarity (familiar versus meaningless items) and stimulus type (letter strings versus pictures) when 58 right-handed healthy subjects who all spoke English as a first language performed 8 different visual processing conditions. The results show condition dependent effects that dissociate the function of superior and inferior parts of vOT at y = −60mm. The superior region is most likely to be part of fusiform region FG4. The inferior region is most likely to be part of fusiform region FG2, see Fig. 3. Our discussion of these results below considers the function of these two regions and the implication of our findings for future studies of vOT function in neurologically normal and clinical populations.

The most striking finding was that activation in superior vOT regions depends on the nature of the task. More attention demanding tasks increase “superior vOT” activation, even when stimuli were held constant. This was demonstrated by increased vOT activation in the most superior part of the occipito-temporal sulcus when participants were attending to unfamiliar stimuli (i.e. Greek letter strings and pictures of nonobjects) and making perceptual matching decisions compared to when the same participants said “1,2,3” in response to the same stimuli – a task that does not require them to pay attention to the perceptual content of the stimuli. Greater superior vOT activation was also found for semantic matching decisions on written words compared to the more familiar task of reading aloud. Although the demand on semantic processing could explain the superior vOT responses to written words, it cannot explain the superior vOT response to perceptual matching decisions on unfamiliar stimuli. We therefore suggest that both task effects can be more parsimoniously explained by increased attention to visual input enhancing activation in superior vOT more than inferior vOT.

The DCM analyses provided further evidence for a dissociation between superior and inferior vOT pathways. While both superior and inferior pathways were found to drive activation in anterior OTs, the relative contribution of the two pathways depended on the nature of the task. In the superior pathway, connectivity strength increased for semantic content during matching but irrespective of semantic content during speaking. In contrast, connectivity strength in the inferior pathway, increased with semantic content during matching and speaking tasks.

Our findings of a dissociation in activation and connectivity in superior and inferior vOT regions have implications for research into the interaction between left vOT and brain regions subserving higher order language function (Price and Devlin, 2011; Woodhead et al., 2013; Perrone-Bertolotti et al., 2017). More specifically, our results lead us to predict that superior and inferior vOT subregions might be differentially sensitive to top down interactions from higher order language and attention areas (Gilbert and Li, 2013). This would complement previous studies showing that different frontal regions interact with posterior and anterior vOT subregions (Mechelli et al., 2005; Seghier and Price, 2013). It would also elaborate more specifically on many emerging studies that illustrate how vOT responses are influenced by higher-order language processing and attention (Seghier et al., 2008; Kawabata Duncan et al., 2013; Schurz et al., 2014; Vandenberghe et al., 2013; Yoncheva et al., 2009; Vogel et al., 2011; Kay and Yeatman, 2017). Our specific prediction is that frontal and parietal regions involved in the control of attention will exert their influence on superior more than inferior vOT subregions. In contrast, frontal and temporal areas involved in linguistic processing will exert influences on inferior more than superior regions. These hypotheses need to be tested in future studies, however, there is already evidence that (i) white matter tracts to vOT vary along the z-axis (Yeatman et al., 2012) with temporal-occipital connections being dorsal to ventral-occipital connections (Rauschecker et al., 2011, 2012). Our DCM findings suggest that the strength of these different dorsal and ventral inputs are modulated by task and stimulus type.

The dissociation of the superior and inferior vOT pathways adds to the growing body of evidence demonstrating that reading is supported by multiple pathways operating in parallel (Iwata, 1984; Ischebeck et al., 2004; Sakurai, 2004; Valdois et al., 2006; Ben-Shachar et al., 2007; Schlaggar and McCandliss, 2007; Kherif et al., 2009; Levy et al., 2009; Rosazza et al., 2009; Wilson et al., 2009; Jobard et al., 2011; Richardson et al., 2011; Seghier et al., 2012; Yvert et al., 2012). Such findings have implications for explaining variability in the symptoms of patients with left vOT damage (Sakurai et al., 2000; Leff et al., 2001; Cohen et al., 2003; Sakurai, 2004; Henry et al., 2005; Gaillard et al., 2006; Newhart et al., 2007; Pyun et al., 2007; Ino et al., 2008; Tsapkini et al., 2011; Seghier et al., 2012). Our study motivates future investigations into how performance differs in patients who have damage to either the superior or inferior vOT. Our knowledge of vOT function may also be enhanced by electrical or magnetic brain stimulation (McKeefry et al., 2009; Duncan et al., 2010) or intracranial recordings (Allison et al., 1994; Nobre and McCarthy, 1995; Jung et al., 2008; Hamamé et al., 2013) directed to superior and inferior vOT subregions. Such studies may eventually lead to more efficient classification of alexia which could have implications for selecting the appropriate course of rehabilitation.

Our findings also have many implications regarding the functional properties of vOT in object perception and recognition. For instance, they suggest that differences in demands on perceptual discrimination need to be considered when designing control/baseline conditions for word or picture stimuli. Such differences in perceptual processing may also explain other previous findings; for instance the response in superior vOT subregions to letter strings versus single letters (James et al., 2005) and the impact of visual crowding on familiar letter processing in vOT (Freeman et al., 2012). Moreover, characterizing differences in activation along the Z-axis may help to understand better the many interactions that vOT entertains with other brain regions. For instance, distinct connectivity profiles of neighbouring regions around the occipito-temporal sulcus have been reported (Yeo et al., 2011), showing a dorsal cluster (at z = −2mm) was correlated with superior parietal cortex and frontal eye field, whereas a ventral cluster (at z = −14mm) was correlated with the inferior parietal lobule (c.f. Figures 30 and 31 of Yeo et al., 2011). These differences in intrinsic connectivity suggest that vOT subregions may participate in distributed networks that are embedded within largely parallel circuits (see discussion in Yeo et al., 2011).

In summary, by examining the influence of multiple experimental variables, our findings show functional differences in superior and inferior vOT activation that have implications for the design and interpretation of visual processing studies. The variability in reading activation along the z-axis was observed here using group level statistics and smoothed data. Therefore it was not a consequence of random inter-subject variability in functional anatomy. Future research is needed to investigate: developmental and retinotopic aspects of vOT function; how involvement of inferior and superior vOT changes with experience; how the inferior and superior vOT subregions are structurally and functionally connected to other brain regions and how this varies over subjects.

## References

[bib1] Allison T., McCarthy G., Nobre A., Puce A., Belger A. (1994). Human extrastriate visual cortex and the perception of faces, words, numbers, and colors. Cerebr. Cortex.

[bib3] Ben-Shachar M., Dougherty R.F., Wandell B.A. (2007). White matter pathways in reading. Curr. Opin. Neurobiol..

[bib4] Binder J.R., Medler D.A., Desai R., Conant L.L., Liebenthal E. (2005). Some neurophysiological constraints on models of word naming. Neuroimage.

[bib5] Binder J.R., Westbury C.F., McKiernan K.A., Possing E.T., Medler D.A. (2005). Distinct brain systems for processing concrete and abstract concepts. J. Cogn. Neurosci..

[bib6] Bolger D.J., Perfetti C.A., Schneider W. (2005). Cross-cultural effect on the brain revisited: universal structures plus writing system variation. Hum. Brain Mapp..

[bib7] Booth J.R., Burman D.D., Meyer J.R., Gitelman D.R., Parrish T.B., Mesulam M.M. (2002). Functional anatomy of intra- and cross-modal lexical tasks. Neuroimage.

[bib8] Bruno J.L., Zumberge A., Manis F.R., Lu Z.L., Goldman J.G. (2008). Sensitivity to orthographic familiarity in the occipito-temporal region. Neuroimage.

[bib9] Cachia A., Roell M., Mangin J.F., Sun Z.Y., Jobert A., Braga L., Houde O., Dehaene S., Borst G. (2018). How interindividual differences in brain anatomy shape reading accuracy. Brain Struct. Funct..

[bib10] Carreiras M., Mechelli A., Estévez A., Price C.J. (2007). Brain activation for lexical decision and reading aloud: two sides of the same coin?. J. Cogn. Neurosci..

[bib11] Caspers J., Zilles K., Eickhoff S.B., Schleicher A., Mohlberg H., Amunts K. (2013). Cytoarchitectonical analysis and probabilistic mapping of two extrastriate areas of the human posterior fusiform gyrus. Brain Struct. Funct..

[bib13] Chee M.W., Weekes B., Lee K.M., Soon C.S., Schreiber A., Hoon J.J., Chee M. (2000). Overlap and dissociation of semantic processing of Chinese characters, English words, and pictures: evidence from fMRI. Neuroimage.

[bib14] Chee M.W., Westphal C., Goh J., Graham S., Song A.W. (2003). Word frequency and subsequent memory effects studied using event-related fMRI. Neuroimage.

[bib15] Cohen L., Lehericy S., Chochon F., Lemer C., Rivaud S., Dehaene S. (2002). Language-specific tuning of visual cortex? Functional properties of the visual word form area. Brain.

[bib16] Cohen L., Martinaud O., Lemer C., Lehéricy S., Samson Y., Obadia M., Slachevsky A., Dehaene S. (2003). Visual word recognition in the left and right hemispheres: anatomical and functional correlates of peripheral alexias. Cerebr. Cortex.

[bib17] Cohen L., Jobert A., Le Bihan D., Dehaene S. (2004). Distinct unimodal and multimodal regions for word processing in the left temporal cortex. Neuroimage.

[bib18] Cohen L., Dehaene S., Vinckier F., Jobert A., Montavont A. (2008). Reading normal and degraded words: contribution of the dorsal and ventral visual pathways. Neuroimage.

[bib19] Danelli L., Berlingeri M., Bottini G., Ferri F., Vacchi L., Sberna M., Paulesu E. (2013). Neural intersections of the phonological, visual magnocellular and motor/cerebellar systems in normal readers: implications for imaging studies on dyslexia. Hum. Brain Mapp..

[bib20] Danelli L., Marelli M., Berlingeri M., Tettamanti M., Sberna M., Paulesu E., Luzzatti C. (2015). Framing effects reveal discrete lexical-semantic and sublexical procedures in reading: an fMRI study. Front. Psychol..

[bib21] Dehaene S., Naccache L., Cohen L., Bihan D.L., Mangin J.F., Poline J.B., Rivière D. (2001). Cerebral mechanisms of word masking and unconscious repetition priming. Nat. Neurosci..

[bib22] Dehaene S., Jobert A., Naccache L., Ciuciu P., Poline J.B., Le Bihan D., Cohen L. (2004). Letter binding and invariant recognition of masked words: behavioral and neuroimaging evidence. Psychol. Sci..

[bib23] Dehaene S., Nakamura K., Jobert A., Kuroki C., Ogawa S., Cohen L. (2010). Why do children make mirror errors in reading? Neural correlates of mirror invariance in the visual word form area. Neuroimage.

[bib24] Destrieux C., Fischl B., Dale A., Halgren E. (2010). Automatic parcellation of human cortical gyri and sulci using standard anatomical nomenclature. Neuroimage.

[bib25] Devlin J.T., Jamison H.L., Gonnerman L.M., Matthews P.M. (2006). The role of the posterior fusiform gyrus in reading. J. Cogn. Neurosci..

[bib26] Duncan K.J., Pattamadilok C., Devlin J.T. (2010). Investigating occipito-temporal contributions to reading with TMS. J. Cogn. Neurosci..

[bib27] Eickhoff S.B., Stephan K.E., Mohlberg H., Grefkes C., Fink G.R., Amunts K., Zilles K. (2005). A new SPM toolbox for combining probabilistic cytoarchitectonic maps and functional imaging data. Neuroimage.

[bib28] Freeman J., Chakravarthi R., Pelli D.G. (2012). Substitution and pooling in crowding. Atten. Percept. Psychophys..

[bib29] Friston K.J., Jezzard P., Turner R. (1994). Analysis of functional MRI time-series. Hum. Brain Mapp..

[bib30] Friston K.J., Harrison L., Penny W. (2003). Dynamic causal modelling. Neuroimage.

[bib31] Gaillard R., Naccache L., Pinel P., Clémenceau S., Volle E., Hasboun D., Dupont S., Baulac M., Dehaene S., Adam C., Cohen L. (2006). Direct intracranial, FMRI, and lesion evidence for the causal role of left inferotemporal cortex in reading. Neuron.

[bib32] Gao Y., Sun Y., Lu C., Ding G., Guo T., Malins J.G., Booth J.R., Peng D., Liu L. (2017). Dynamic spatial organization of the occipito-temporal word form area for second language processing. Neuropsychologia.

[bib33] Gauthier I., Tarr M.J., Moylan J., Skudlarski P., Gore J.C., Anderson A.W. (2000). The fusiform "face area" is part of a network that processes faces at the individual level. J. Cogn. Neurosci..

[bib34] Gilbert C.D., Li W. (2013). Top-down influences on visual processing. Nat. Rev. Neurosci..

[bib35] Guo Y., Burgund E.D. (2010). Task effects in the mid-fusiform gyrus: a comparison of orthographic, phonological, and semantic processing of Chinese characters. Brain Lang..

[bib36] Hamamé C.M., Szwed M., Sharman M., Vidal J.R., Perrone-Bertolotti M., Kahane P., Bertrand O., Lachaux J.P. (2013). Dejerines' reading area revisited with intracranial-EEG: selective responses to letter-strings. Neurology.

[bib37] Hayashi A., Okamoto Y., Yoshimura S., Yoshino A., Toki S., Yamashita H., Matsuda F., Yamawaki S. (2014). Visual imagery while reading concrete and abstract Japanese kanji words: an fMRI study. Neurosci. Res..

[bib38] Henry C., Gaillard R., Volle E., Chiras J., Ferrieux S., Dehaene S., Cohen L. (2005). Brain activations during letter-by-letter reading: a follow-up study. Neuropsychologia.

[bib40] Ino T., Tokumoto K., Usami K., Kimura T., Hashimoto Y., Fukuyama H. (2008). Longitudinal fMRI study of reading in a patient with letter-by-letter reading. Cortex.

[bib41] Ischebeck A., Indefrey P., Usui N., Nose I., Hellwig F., Taira M. (2004). Reading in a regular orthography: an FMRI study investigating the role of visual familiarity. J. Cogn. Neurosci..

[bib43] Iwata M. (1984). Kanji versus Kana: neuropsychological correlates of the Japanese writing system. Trends Neurosci..

[bib44] James K.H., James T.W., Jobard G., Wong A.C., Gauthier I. (2005). Letter processing in the visual system: different activation patterns for single letters and strings. Cognit. Affect Behav. Neurosci..

[bib45] Jobard G., Crivello F., Tzourio-Mazoyer N. (2003). Evaluation of the dual route theory of reading: a metanalysis of 35 neuroimaging studies. Neuroimage.

[bib46] Jobard G., Vigneau M., Simon G., Tzourio-Mazoyer N. (2011). The weight of skill: interindividual variability of reading related brain activation patterns in fluent readers. J. Neurolinguistics.

[bib47] Josse G., Seghier M.L., Kherif F., Price C.J. (2008). Explaining function with anatomy: language lateralization and corpus callosum size. J. Neurosci..

[bib48] Jung J., Mainy N., Kahane P., Minotti L., Hoffmann D., Bertrand O., Lachaux J.P. (2008). The neural bases of attentive reading. Hum. Brain Mapp..

[bib50] Kao C.H., Chen D.Y., Chen C.C. (2010). The inversion effect in visual word form processing. Cortex.

[bib51] Kawabata Duncan K.J., Twomey T., Parker Jones O., Seghier M.L., Haji T., Sakai K., Price C.J., Devlin J.T. (2013). Inter-and intrahemispheric connectivity differences when reading Japanese Kanji and Hiragana. Cerebr. Cortex.

[bib52] Kay K.N., Yeatman J.D. (2017). Bottom-up and top-down computations in word- and face-selective cortex. Elife.

[bib53] Kherif F., Josse G., Seghier M.L., Price C.J. (2009). The main sources of inter-subject variability in neuronal activation for reading aloud. J. Cogn. Neurosci..

[bib54] Kherif F., Josse G., Price C.J. (2011). Automatic top-down processing explains common left occipito-temporal responses to visual words and objects. Cerebr. Cortex.

[bib55] Kiehl K.A., Liddle P.F., Smith A.M., Mendrek A., Forster B.B., Hare R.D. (1999). Neural pathways involved in the processing of concrete and abstract words. Hum. Brain Mapp..

[bib56] Kim H., Bernasconi N., Bernhardt B., Colliot O., Bernasconi A. (2008). Basal temporal sulcal morphology in healthy controls and patients with temporal lobe epilepsy. Neurology.

[bib57] Kronbichler M., Hutzler F., Wimmer H., Mair A., Staffen W., Ladurner G. (2004). The visual word form area and the frequency with which words are encountered: evidence from a parametric fMRI study. Neuroimage.

[bib58] Kronbichler M., Bergmann J., Hutzler F., Staffen W., Mair A., Ladurner G., Wimmer H. (2007). Taxi vs. taksi: on orthographic word recognition in the left ventral occipitotemporal cortex. J. Cogn. Neurosci..

[bib59] Kronbichler M., Klackl J., Richlan F., Schurz M., Staffen W., Ladurner G., Wimmer H. (2009). On the functional neuroanatomy of visual word processing: effects of case and letter deviance. J. Cogn. Neurosci..

[bib60] Leff A.P., Crewes H., Plant G.T., Scott S.K., Kennard C., Wise R.J.S. (2001). The functional anatomy of single-word reading in patients with hemianopic and pure alexia. Brain.

[bib61] Lerma-Usabiaga G., Carreiras M., Paz-Alonso P.M. (2018). Converging evidence for functional and structural segregation within the left ventral occipitotemporal cortex in reading. Proc. Natl. Acad. Sci. U. S. A..

[bib62] Levy J., Pernet C., Treserras S., Boulanouar K., Aubry F., Démonet J.F., Celsis P. (2009). Testing for the dual-route cascade reading model in the brain: an fMRI effective connectivity account of an efficient reading style. PLoS One.

[bib63] Lorenz S., Weiner K.S., Caspers J., Mohlberg H., Schleicher A., Bludau S., Eickhoff S.B., Grill-Spector K., Zilles K., Amunts K. (2017). Two new cytoarchitectonic areas on the human mid-fusiform gyrus. Cerebr. Cortex.

[bib64] Ma L., Jiang Y., Bai J., Gong Q., Liu H., Chen H.C., He S., Weng X. (2011). Robust and task-independent spatial profile of the visual word form activation in fusiform cortex. PLoS One.

[bib65] Malikovic A., Vucetic B., Milisavljevic M., Tosevski J., Sazdanovic P., Milojevic B., Malobabic S. (2012). Occipital sulci of the human brain: variability and morphometry. Anat. Sci. Int..

[bib66] McKeefry D.J., Gouws A., Burton M.P., Morland A.B. (2009). The noninvasive dissection of the human visual cortex: using FMRI and TMS to study the organization of the visual brain. Neuroscientist.

[bib67] Mechelli A., Gorno-Tempini M.L., Price C.J. (2003). Neuroimaging studies of word and pseudoword reading: consistencies, inconsistencies, and limitations. J. Cogn. Neurosci..

[bib68] Mechelli A., Crinion J.T., Long S., Friston K.J., Lambon Ralph M.A., Patterson K., McClelland J.L., Price C.J. (2005). Dissociating reading processes on the basis of neuronal interactions. J. Cogn. Neurosci..

[bib69] Mongelli V., Dehaene S., Vinckier F., Peretz I., Bartolomeo P., Cohen L. (2017). Music and words in the visual cortex: the impact of musical expertise. Cortex.

[bib70] Nestor A., Behrmann M., Plaut D.C. (2012). The neural basis of visual word form processing: a multivariate investigation. Cerebr. Cortex.

[bib71] Newhart M., Ken L., Kleinman J.T., Heidler-Gary J., Hillis A.E. (2007). Neural networks essential for naming and word comprehension. Cogn. Behav. Neurol..

[bib72] Nobre A.C., McCarthy G. (1995). Language-related field potentials in the anterior-medial temporal lobe: II. Effects of word type and semantic priming. J. Neurosci..

[bib73] Nosarti C., Mechelli A., Green D.W., Price C.J. (2010). The impact of second language learning on semantic and nonsemantic first language reading. Cerebr. Cortex.

[bib74] Ono M., Kubic S., Abernathey C.D. (1990). Atlas of the Cerebral Sulci.

[bib75] Peng D.L., Xu D., Jin Z., Luo Q., Ding G.S., Perry C., Zhang L., Liu Y. (2003). Neural basis of the non-attentional processing of briefly presented words. Hum. Brain Mapp..

[bib76] Perrone-Bertolotti M., Kauffmann L., Pichat C., Vidal J.R., Baciu M. (2017). Effective connectivity between ventral occipito-temporal and ventral inferior frontal cortex during lexico-semantic processing. A dynamic causal modeling study. Front. Hum. Neurosci..

[bib1a] Price C.J. (2012). A review and synthesis of the first 20 years of PET and fMRI studies of heard speech, spoken language and reading. NeuroImage.

[bib77] Price C.J., Mechelli A. (2005). Reading and reading disturbance. Curr. Opin. Neurobiol..

[bib78] Price C.J., Devlin J.T. (2011). The Interactive Account of ventral occipito-temporal contributions to reading. Trends Cognit. Sci..

[bib79] Purcell J.J., Napoliello E.M., Eden G.F. (2011). A combined fMRI study of typed spelling and reading. Neuroimage.

[bib80] Pyun S.B., Sohn H.J., Jung J.B., Nam K. (2007). Differential reorganization of fusiform gyrus in two types of alexia after stroke. Neurocase.

[bib81] Quinn C., Taylor J.S.H., Davis M.H. (2017). Learning and retrieving holistic and componential visual-verbal associations in reading and object naming. Neuropsychologia.

[bib82] Rauschecker A.M., Bowen R.F., Parvizi J., Wandell B.A. (2012). Position sensitivity in the visual word form area. Proc. Natl. Acad. Sci. U. S. A..

[bib83] Rauschecker A.M., Bowen R.F., Perry L.M., Kevan A.M., Dougherty R.F., Wandell B.A. (2011). Visual feature-tolerance in the reading network. Neuron.

[bib84] Richardson F.M., Seghier M.L., Leff A.P., Thomas M.S.C., Price C.J. (2011). Multiple routes from occipital to temporal cortices during reading. J. Neurosci..

[bib85] Rosa M.J., Friston K., Penny W. (2012). Post-hoc selection of dynamic causal models. J. Neurosci. Methods.

[bib86] Rosazza C., Cai Q., Minati L., Paulignan Y., Nazir T.A. (2009). Early involvement of dorsal and ventral pathways in visual word recognition: an ERP study. Brain Res..

[bib87] Sakurai Y., Takeuchi S., Takada T., Horiuchi E., Nakase H., Sakuta M. (2000). Alexia caused by a fusiform or posterior inferior temporal lesion. J. Neurol. Sci..

[bib88] Sakurai Y. (2004). Varieties of alexia from fusiform, posterior inferior temporal and posterior occipital gyrus lesions. Behav. Neurol..

[bib89] Sandak R., Mencl W.E., Frost S.J., Rueckl J.G., Katz L., Moore D.L., Mason S.A., Fulbright R.K., Constable R.T., Pugh K.R. (2004). The neurobiology of adaptive learning in reading: a contrast of different training conditions. Cognit. Affect Behav. Neurosci..

[bib90] Schlaggar B.L., McCandliss B.D. (2007). Development of neural systems for reading. Annu. Rev. Neurosci..

[bib91] Schurz M., Sturm D., Richlan F., Kronbichler M., Ladurner G., Wimmer H. (2010). A dual-route perspective on brain activation in response to visual words: evidence for a length by lexicality interaction in the visual word form area (VWFA). Neuroimage.

[bib92] Schurz M., Kronbichler M., Crone J., Richlan F., Klackl J., Wimmer H. (2014). Top-down and bottom-up influences on the left ventral occipito-temporal cortex during visual word recognition: an analysis of effective connectivity. Hum. Brain Mapp..

[bib93] Schuster S., Hawelka S., Richlan F., Ludersdorfer P., Hutzler F. (2015). Eyes on words: a fixation-related fMRI study of the left occipito-temporal cortex during self-paced silent reading of words and pseudowords. Sci. Rep..

[bib94] Seghier M.L., Lee H.L., Schofield T., Ellis C.L., Price C.J. (2008). Inter-subject variability in the use of two different neuronal networks for reading aloud familiar words. Neuroimage.

[bib95] Seghier M.L., Zeidman P., Neufeld N.H., Leff A.P., Price C.J. (2010). Identifying abnormal connectivity in patients using Dynamic Causal Modelling of fMRI responses. Front. Syst. Neurosci..

[bib96] Seghier M.L., Price C.J. (2011). Explaining left lateralization for words in the ventral occipitotemporal cortex. J. Neurosci..

[bib97] Seghier M.L., Neufeld N.H., Zeidman P., Leff A.P., Mechelli A., Nagendran A., Riddoch J.M., Humphreys G.W., Price C.J. (2012). Reading without the left ventral occipito-temporal cortex. Neuropsychologia.

[bib98] Seghier M.L., Price C.J. (2013). Dissociating frontal regions that co-lateralize with different ventral occipitotemporal regions during word processing. Brain Lang..

[bib99] Simons J.S., Koutstaal W., Prince S., Wagner A.D., Schacter D.L. (2003). Neural mechanisms of visual object priming: evidence for perceptual and semantic distinctions in fusiform cortex. Neuroimage.

[bib101] Stephan K.E., Penny W.D., Moran R.J., den Ouden H.E., Daunizeau J., Friston K.J. (2010). Ten simple rules for dynamic causal modeling. Neuroimage.

[bib102] Stevens W.D., Kravitz D.J., Peng C.S., Tessler M.H., Martin A. (2017). Privileged functional connectivity between the visual word form area and the language system. J. Neurosci..

[bib103] Sussman B.L., Reddigari S., Newman S.D. (2018). The impact of inverted text on visual word processing: an fMRI study. Brain Cogn..

[bib104] Szwed M., Qiao E., Jobert A., Dehaene S., Cohen L. (2014). Effects of literacy in early visual and occipitotemporal areas of Chinese and French readers. J. Cogn. Neurosci..

[bib105] Taylor J.S.H., Rastle K., Davis M.H. (2013). Can cognitive models explain brain activation during word and pseudoword reading? A meta-analysis of 36 neuroimaging studies. Psychol. Bull..

[bib106] Thesen T., McDonald C.R., Carlson C., Doyle W., Cash S., Sherfey J., Felsovalyi O., Girard H., Barr W., Devinsky O., Kuzniecky R. (2012). Sequential then interactive processing of letters and words in the left fusiform gyrus. Nat. Commun..

[bib107] Tsapkini K., Vindiola M., Rapp B. (2011). Patterns of brain reorganization subsequent to left fusiform damage: fMRI evidence from visual processing of words and pseudowords, faces and objects. Neuroimage.

[bib108] Twomey T., Duncan K.J.K., Hogan J.S., Morita K., Umeda K., Sakai K., Devlin J.T. (2013). Dissociating visual form from lexical frequency using Japanese. Brain Lang..

[bib110] Valdois S., Carbonnel S., Juphard A., Baciu M., Ans B., Peyrin C., Segebarth C. (2006). Polysyllabic pseudo-word processing in reading and lexical decision: converging evidence from behavioral data, connectionist simulations and functional MRI. Brain Res..

[bib111] Vandenberghe R., Wang Y., Nelissen N., Vandenbulcke M., Dhollander T., Sunaert S., Dupont P. (2013). The associative-semantic network for words and pictures: effective connectivity and graph analysis. Brain Lang..

[bib112] Vinckier F., Dehaene S., Jobert A., Dubus J.P., Sigman M., Cohen L. (2007). Hierarchical coding of letter strings in the ventral stream: dissecting the inner organization of the visual word-form system. Neuron.

[bib113] Vogel A.C., Miezin F.M., Petersen S.E., Schlaggar B.L. (2011). The putative visual word form area is functionally connected to the dorsal attention network. Cerebr. Cortex.

[bib114] Wang X., Xu Y., Wang Y., Zeng Y., Zhan J., Ling Z., Bi Y. (2018). Representational similarity analysis reveals task-dependent semantic influence of the visual word form area. Sci. Rep..

[bib115] Weiner K.S., Barnett M.A., Lorenz S., Caspers J., Stigliani A., Amunts K., Zilles K., Fischl B., Grill-Spector K. (2017). The cytoarchitecture of domain-specific regions in human high-level visual cortex. Cerebr. Cortex.

[bib116] Weiss Y., Booth J.R. (2017). Neural correlates of the lexicality effect in children. Brain Lang..

[bib117] Wilson S.M., Brambati S.M., Henry R.G., Handwerker D.A., Agosta F., Miller B.L., Wilkins D.P., Ogar J.M., Gorno-Tempini M.L. (2009). The neural basis of surface dyslexia in semantic dementia. Brain.

[bib118] Wimmer H., Ludersdorfer P., Richlan F., Kronbichler M. (2016). Visual experience shapes orthographic representations in the visual word form area. Psychol. Sci..

[bib119] Woodhead Z.V., Barnes G.R., Penny W., Moran R., Teki S., Price C.J., Leff A.P. (2013). Reading front to back: MEG evidence for early feedback effects during word recognition. Cerebr. Cortex.

[bib120] Woollams A.M., Silani G., Okada K., Patterson K., Price C.J. (2011). Word or word-like? Dissociating orthographic typicality from lexicality in the left occipito-temporal cortex. J. Cogn. Neurosci..

[bib121] Wright N.D., Mechelli A., Noppeney U., Veltman D.J., Rombouts S.A., Glensman J., Haynes J.D., Price C.J. (2008). Selective activation around the left occipito-temporal sulcus for words relative to pictures: individual variability or false positives?. Hum. Brain Mapp..

[bib122] Xu M., Wang T., Chen S., Fox P.T., Tan L.H. (2015). Effective connectivity of brain regions related to visual word recognition: an fMRI study of Chinese reading. Hum. Brain Mapp..

[bib123] Xue G., Chen C., Jin Z., Dong Q. (2006). Language experience shapes fusiform activation when processing a logographic artificial language: an fMRI training study. Neuroimage.

[bib124] Xue G., Poldrack R.A. (2007). The neural substrates of visual perceptual learning of words: implications for the visual word form area hypothesis. J. Cogn. Neurosci..

[bib125] Yarkoni T., Speer N.K., Balota D.A., McAvoy M.P., Zacks J.M. (2008). Pictures of a thousand words: investigating the neural mechanisms of reading with extremely rapid event-related fMRI. Neuroimage.

[bib126] Yeatman J.D., Rauschecker A.M., Wandell B.A. (2012). Anatomy of the visual word form area: adjacent cortical circuits and long-range white matter connections. Brain Lang..

[bib127] Yeo B.T., Krienen F.M., Sepulcre J., Sabuncu M.R., Lashkari D., Hollinshead M., Roffman J.L., Smoller J.W., Zöllei L., Polimeni J.R., Fischl B. (2011). The organization of the human cerebral cortex estimated by intrinsic functional connectivity. J. Neurophysiol..

[bib128] Yoncheva Y.N., Zevin J.D., Maurer U., McCandliss B.D. (2009). Auditory selective attention to speech modulates activity in the visual word form area. Cerebr. Cortex.

[bib129] Yvert G., Perrone-Bertolotti M., Baciu M., David O. (2012). Dynamic causal modeling of spatiotemporal integration of phonological and semantic processes: an electroencephalographic study. J. Neurosci..

